# Enhancing bioavailability of natural extracts for nutritional applications through dry powder inhalers (DPI) spray drying: technological advancements and future directions

**DOI:** 10.3389/fnut.2023.1190912

**Published:** 2023-07-05

**Authors:** Bo Wang, Jia Xiang, Binsheng He, Songwen Tan, Wenhu Zhou

**Affiliations:** ^1^Academician Workstation, Changsha Medical University, Changsha, China; ^2^Xiangya School of Pharmaceutical Sciences, Central South University, Changsha, Hunan, China

**Keywords:** natural ingredients, spray drying, dry powder inhalation, factors influencing DPI, application of natural extracts

## Abstract

Natural ingredients have many applications in modern medicine and pharmaceutical projects. However, they often have low solubility, poor chemical stability, and low bioavailability *in vivo*. Spray drying technology can overcome these challenges by enhancing the properties of natural ingredients. Moreover, drug delivery systems can be flexibly designed to optimize the performance of natural ingredients. Among the various drug delivery systems, dry powder inhalation (DPI) has attracted much attention in pharmaceutical research. Therefore, this review will focus on the spray drying of natural ingredients for DPI and discuss their synthesis and application.

## Graphical Abstract

Nutrition application process via dry powder inhaler (DPI) spray drying. 
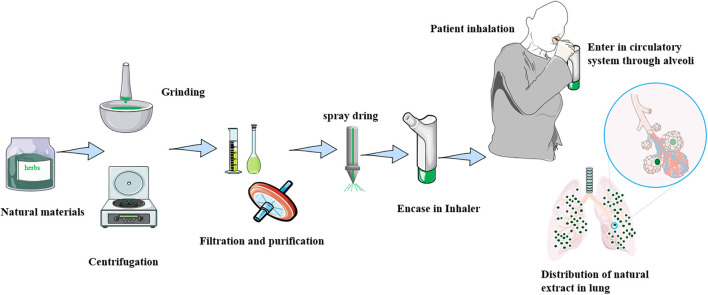

## 1. Introduction

Nowadays, natural ingredients are becoming more and more important in medical research because they have a wide range of clinical benefits. They have rich pharmacological effects, such as anti-cancer, anti-oxidation, lowering blood lipid, inducing autophagy and reducing the side effects of drug therapy (1–4). However, at present, natural ingredients have many shortcomings, such as chemical instability, low solubility and low system bioavailability, which need to be solved by various technical means in order to give full play to their potential in modern medicine (5, 6).

**FIGURE 1:**
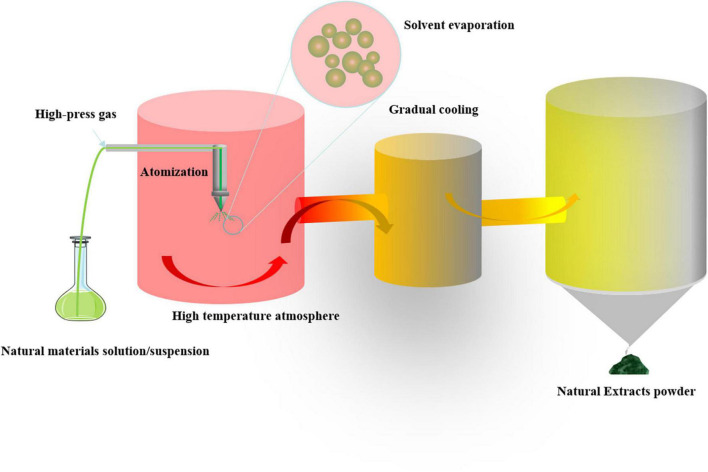
Spray drying process of natural material.

In order to solve these problems, researchers have developed various methods and found that spray drying technology may be a potential solution. Spray drying technology can accurately control the particle size of drugs, improve the dispersion of drugs in the lung, and reduce drug deposition, thus achieving better therapeutic effect. Spray drying technology can transform crystalline drug into an amorphous solid dispersion, which shows a more disordered molecular structure and is more soluble. Amorphous drug has a high energy state, compared with crystalline drug, its saturated solubility and dissolution rate are significantly improved. Spray drying technology can produce dry powder pharmaceutical preparations with good stability. Due to the presence of polymer, amorphous drug can be effectively stabilized, preventing it from crystallizing or degrading during storage. Because of its unique properties, spray-drying is widely used in pharmaceutical preparations. It can accurately control the particle size of drugs, improve the dispersion of drugs in the lung, and reduce drug deposition, so as to achieve better therapeutic effect (7–10). The principle of spray drying is shown in Figure 1. Meanwhile, spray drying technology can significantly improve the solubility, permeability and stability of natural products. For example, the microencapsulation system prepared by spray drying improves the *in vitro* dissolution and permeation rates of silymarin, enhancing its bioactivity after oral administration. This system effectively preserves the antioxidant activity of silymarin and does not compromise the anti-inflammatory properties of the original extract. It seems that the anthocyanin polyphenols treated by spray drying technology have a higher retention rate and their color characteristics are also maintained. At the same time, it protects phenolic acids and flavonoids from thermal degradation (11, 12). The microencapsulated components of epicatechin and glycosylated quercetin have been proved to protect phenolic acids and flavonoids from thermal degradation in an environment simulating gastrointestinal conditions (13), and the microencapsulated components of epicatechin and glycosylated quercetin remain amorphous after long-term storage, which ensures their long-term solubility and bioavailability *in vitro* (14). These findings indicate that the use of spray drying techniques can effectively improve the physical properties of natural ingredients and be further applied to clinical practice for the preparation of various formulations, including the development and application of DPI.

**FIGURE 2:**
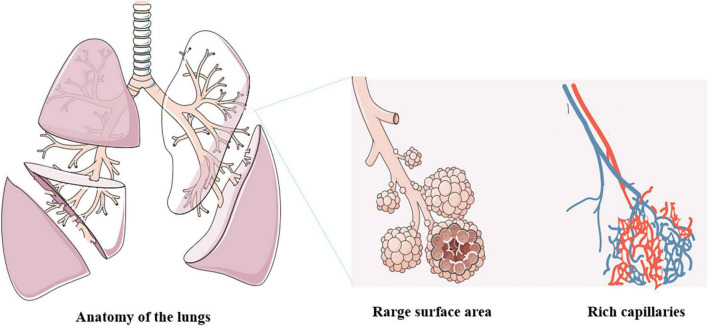
The anatomy of the lungs.

The pulmonary drug delivery route provides a direct and effective way to deliver the drug to the systemic circulation, which has many advantages: (1) It can deliver the drug directly to the systemic circulation without passing through the liver, thus avoiding the first-pass effect and improving bioavailability (15); (2) It can take advantage of the anatomical and physiological characteristics of the lung, including large surface area, abundant capillaries and relatively few metabolic reactions, to provide an ideal place for drug transport (Figure 2) (16, 17); (3) It can design suitable DPI pulmonary drug delivery systems according to the drug’s absorption, distribution, metabolism, excretion and toxicity characteristics (ADMET) (18–21); (4) It can target pulmonary diseases, such as asthma, chronic obstructive pulmonary disease (COPD), pulmonary fibrosis and COVID-19, and deliver the drug directly to the lung tissue, achieving local effective results; (5) It can significantly reduce the dosage of the drug and reduce the adverse reactions caused by drug distribution in other parts, compared with other delivery methods (22–26); (6) It can also be used for the treatment and prevention of other systemic diseases, such as diabetes, cancer, osteoporosis and so on. Therefore, for some special pulmonary diseases, the advantages of pulmonary drug delivery are irreplaceable. At the same time, the dry powder inhalation pulmonary drug delivery method has unique advantages in the treatment and prevention of other systemic diseases.

Combined with the characteristics of natural ingredients, this paper mainly introduces the application of spray drying technology in DPIs. Spray drying technology can improve the bioavailability, stability, and solubility of drugs (10, 11, 27–29). Spray drying technology has a broad prospect in the production of dry powder inhalers for natural medicine. Spray drying can not only alleviate the problem of natural ingredients but also greatly improve their efficacy and value. In summary, this paper aims to introduce spray-drying natural ingredients for DPIs and recent research.

## 2. Composition of the DPIs of natural ingredient

### 2.1. Composition of natural component DPI

Dry powder inhalation consists of natural ingredients (such as lactose, starch, and cellulose) and natural extracts with therapeutic properties. Its composition may vary depending on the formula of the drugs and the delivery device. Generally, natural ingredients play an important role in the safety and effectiveness of DPI as a pulmonary drug delivery method. The drugs containing natural ingredients differ according to the treatment purpose (30–33).

Natural components are an important part of drug research and development. Since 1981, about half of the small molecule drugs approved by the US Food and Drug Administration (FDA) have come from natural sources. With the progress of science and technology, researchers pay more and more attention to the pharmacological activities of trace, insoluble, and unstable components in natural products, which used to be difficult to obtain. The advances in infrared (IR), ultraviolet-visible (UV), mass spectrometry (MS), and nuclear magnetic resonance (NMR) spectroscopy can determine the structure of active substances in natural components and promote the study of their pharmacological activities. In addition, various chromatographic separation techniques have been used to extract and purify natural components, thus allowing the mass production of trace amounts of these substances and developing them into effective treatment methods for diseases. Natural ingredients are often used in anti-cancer, anti-oxidation, anti-inflammatory, and antibacterial therapy, making their pharmacological activities an indispensable part of modern medicine. Some common natural chemicals are listed in Table 1.

**TABLE 1:** Natural chemicals may use in DPIs.

	Compound	Source	Effects
Herbs	Morphine and codeine	Opium poppy	Analgesic, pain relief (34).
	Caffeine	Coffee beans	Analgesic, headache relief, neuropathic pain relief, diuretic (35).
	Cannabinoid	Marijuana	Analgesic, nausea relief, vomiting relief (36).
	Willow bark extract	Willow	Anti-inflammatory, pain relief (37).
	Flavonoids (quercetin, rutin)	Various plants	Anti-inflammatory, reduce allergic reactions, protect heart health (38).
	Polyphenols (tea polyphenol, grape seed extract)	Tea, grapes	Antioxidant, reduce oxidative damage from free radicals (39)
	Vitamins	Various fruits and vegetables	Antioxidant, reduce oxidative damage from free radicals and so on (40, 41).
	Echinacea	Echinacea	Anticancer, inhibit cancer cell growth (42).
	Lignan	Flaxseed	Anticancer, inhibit cancer cell growth (43).
	Saponins	Pueraria lobata, schisandra chinensis	Hypoglycemic, reduce blood glucose levels (44).
	Triterpenes (cinnamyl, ilex)	Cinnamon, ilex	Lipid-lowering, reduce cholesterol and triglyceride levels (45).
	Polysaccharides (ganoderma polysaccharides, cordyceps polysaccharides)	Ganoderma, cordyceps	Immunomodulatory, regulate immune system function, enhance immunity (46).
	Berberine	Rhizoma Coptidis	Antibacterial, inhibit bacterial and viral growth (47).
	Allicin	Garlic	Antibacterial, antiviral, inhibit bacterial and viral growth (48).
	Dandelion	Dandelion	Antibacterial, inhibit bacterial and fungal growth (49).
	Flavanones (grape naringin, citric acid)	Citrus fruits	Diuretic, promote urination and increase urine volume (50).
Animals	Cerebrospinal fluid extract	Brain	Neuroprotective, protect nervous system health (51).
	Fish oil	Fish	Neuroprotective, promote nerve cell regeneration, protect nervous system health, rich in omega-3 fatty acids (52).
	Insulin	Extracted from the pancreas	Treating diabetes (53).
	Antibody	Immune system of mammals	Anticancer, immunological diseases (54).
	Heparin	Liver and lungs of animals	Thrombosis and cardiovascular diseases (55).
Minerals	Since most substances of mineral origin are not suitable for inhalation, they are not cited		

In addition, the research progress of some common pharmacological actions of dry powder inhalers are also listed.

#### 2.1.1. Antitumor effect

In the field of tumor research, natural products and their derivatives play an important role in the research and development of small-molecule anticancer drugs. From the 1940s to the present, 48.6% of 175 small-molecule drugs with anticancer activity were directly derived from natural products (56). Examples of such drugs include vinca alkaloids and taxanes, which target microtubules, inhibit mitosis, lead to cell cycle arrest and tumor cell death, and finally achieve anti-cancer effects. A lot of research shows that the natural ingredients in DPI can effectively treat various kinds of cancer. For example, Adel designed curcumin proliposomes by nano-spray-drying, which improved the delivery concentration of curcumin in lung tissue and overcame its shortcomings such as poor water solubility and low oral bioavailability. Curcumin proliposomes effectively inhibited the growth of lung tumor cells, including 549 cells, and showed a better anticancer effect than oral drugs (57).

#### 2.1.2. Anti-inflammatory effect

Many natural compounds, such as oridonin (58), verbascoside (59), and quercetin (60), have anti-inflammatory activities. For example, resveratrol can regulate inflammatory reactions by inhibiting the synthesis and release of pro-inflammatory mediators, thus showing good anti-inflammatory activity. However, the low bioavailability of resveratrol restricts its clinical application. To solve this problem, researchers developed resveratrol DPI. By spray-drying resveratrol particles to make their diameter less than 5 microns, they can enhance their transport across cell membranes and improve their ability to reach target organs, thus producing more effective anti-inflammatory effects (61, 62).

#### 2.1.3. Antioxidant effect

Resveratrol (63), carotenoids (64), and ginkgolide (65) are the antioxidant substances commonly found in natural active extracts, which can inhibit or prevent the initiation and diffusion of free radical chain reactions, reduce the concentration of free radicals, and slow down cellular aging. A study showed that spray-dried resveratrol has the same antioxidant properties as vitamin C (62). Meanwhile, fluoxetine, a natural antioxidant found in flavonoid plant polyphenols (66), also possesses antioxidant activity. In addition, Mohtar et al. prepared a complex of fluoxetine and cyclodextrin for dry powder inhalation and compared it with ascorbic acid. They found that the IC50 values for free fluoxetine and fluoxetine-cyclodextrin complex solutions were significantly lower (p 0.05) (67), indicating that fluoxetine has excellent antioxidant activity for dry powder inhalation.

#### 2.1.4. Antibacterial effect

From 1981 to 2019, about 50% of all drugs approved by the FDA originated from natural products, and many of them had natural antibacterial functions (68), such as baicalin (69), berberine (70), and evodiamine (71). Besides, research also proved that inhaling andrographolide (AG) dry powder is effective in reducing pro-inflammatory cytokines, regulating the immune response, and downregulating inflammation. AG also showed antibacterial activity and was found to be 10 times more effective than penicillin in the treatment of *S. aureus* pneumonia. Therefore, it has the potential for pulmonary administration in the treatment of bacterial pneumonia.

### 2.2. Carriers and additives

Dry powder inhalation drugs are micronized, so they need to add carriers and additives to improve their fluidity and dispersibility (72–75), and lactose is the most commonly used carrier, as well as the only DPI carrier approved by the FDA (76), but it also has drawbacks and limitations, such as not beingsuitable for diabetic patients and binding too tightly with some drugs, affecting release (77–79). Therefore, some studies have explored other alternative carriers, such as mannitol and erythritol (80–83). The functions of carriers and additives are diverse, including increasing stability, enhancing absorption, improving the respiratory tract environment, etc. Table 2 lists some common functions.

**TABLE 2:** Function of the carriers and additives.

Character	Description	Function	Example
Shape	Different particle shapes encounter varying degrees of resistance during drug transport, affecting the deposition site in the lung. Carriers with high elongation (ER) have lower bulk density [D(b)], tap density [D(t)], higher porosity (P), and reduced fluidity (84). Decreased carrier fluidity leads to more drug loss and deposition in the throat, indicating poor DPI performance.	Varying carrier shapes affect drug deposition.	Kaialy et al. compared crystallized mannitol: five different transitions of lactose corresponding to α-mannitol, α-lactose monohydrate, β-lactose, 5α-/3β-lactose and 4α-/1β-lactose. The products are significantly different in shape and density, and *in vitro* deposition assessment showed that the crystalline carrier produced a more efficient delivery of salbutamol sulfate (85).
Surface roughness	Rough carrier surface increases drug load and allows small drug particles to adhere, facilitating aggregation. Surface roughness also affects drug shape. Coarse spherical carrier particles improve drug dispersion and yield the highest fine particle delivery (FPY) to the deep lung (86).	Rough carrier surface enhances drug load and dispersion.	The processing of lactose carrier particles by roller pressing is very beneficial in improving DPI properties by increasing the surface roughness on a macro scale (87).
Particle size	Carrier particle size is important, although its impact on drug deposition after inhalation is unclear. Smaller particles increase drug delivery to the lungs via dry powder inhalers, but reduce particle fluidity. Using larger particle size carriers (e.g., mannitol) improves fluidity, dosage uniformity, and reduces drug deposition in the throat (88).	Optimal particle size depends on drug characteristics.	DPI prepared from mannitol with larger particle size exhibits narrower size distribution, higher bulk and vibrational densities, lower porosity and better flowability, resulting in optimized and stable DPI formulations with excellent physicochemical and pharmaceutical properties (88).
Lubrication	Lubricants with anti-adhesion and anti-friction properties reduce particle agglomeration, interparticle friction, and improve powder fluidity, leading to enhanced lung deposition and drug dispersion (89, 90). Addition of glyceryl monostearate (MGS) to budesonide (BDS) treatment results in a smoother particle surface, lowering cohesion and improving BDS inhalation characteristics (91).	Lubricants enhance powder fluidity and dispersion.	The magnesium stearate coating and the l-leucine coating not only reduce the surface energy, but also create a rough particle surface, reducing the contact area between particles and increasing the stability and flow of the DPI formulation (90).
Stability	Addition of substances with high glass transition temperature (Tg) forms an amorphous glass matrix, increasing preparation stability (92). Common stabilizers for DPI include amphetamine acid (93) and mannitol (94).	Stabilizers with high Tg improve DPI stability.	Seaweed and cottonseed sugars, which significantly increase the glass transition temperature (Tgs) of proteins and are good stabilizers of protein DPI (92).
Solubilization	Poorly soluble drugs in DPI may exhibit low efficacy. Acidic substances can be added to create an acidic microenvironment, enhancing drug solubility, dissolution, and DPI performance (95). Mixed organic solvents or solubilizers can be used to improve water solubility of drugs (96, 97).	Acidic environment or solvents enhance drug solubility in DPI.	Carriers such as lactose can rapidly lose water with insoluble crystalline drugs after spray drying to form, amorphous solid dispersions, significantly increasing the solubility of the active pharmaceutical ingredients (API), while the addition of solubilizers can also achieve a bulking effect (10).

## 3. Spray-dried DPI

Spray drying is used to atomize liquid materials (e.g., solutions, suspensions, emulsions, etc.). By atomizing the liquid into fine particles and then contacting them with a stream of hot air, the liquid is rapidly evaporated to form a dry powder or granule. The process of spray drying usually includes spraying, drying, powder separation, and collection steps. After spraying, the liquid particles enter the preheating drying chamber. In the high-temperature hot air flow, the water quickly evaporates and the particles gradually decrease, reaching a dry state. The temperature and humidity of the drying chamber can be adjusted according to the characteristics of the material and the target powder (98).

Spray drying technology has been used in traditional pharmaceutical industries for a long time to manufacture pharmaceutical products with specific physical and chemical properties. The product is composed of porous, uniform particles (99) with tiny and precise sizes, which is beneficial to the quality control of DPI. In spray drying, the final state of the product can be adjusted by adjusting various parameters of spray drying, which have the greatest impact (100–102): (1) Solvent selection: The choice of solvent has a significant impact on the change in solubility and bioavailability. The appropriate solvent can fully dissolve the target natural extract and obtain a higher proportion of amorphous products. (2) Nozzle diameter selection: nozzle diameter is positively correlated with product diameter; (3) Solid content of feed liquid: It affects the viscosity, surface tension, and drying rate of feed, as well as the yield, particle size, and morphology of powder. Generally speaking, higher solid content leads to larger, more spherical particles and a higher yield. (4) Inlet air temperature and outlet air temperature refer mainly to the temperature of the drying chamber. Generally speaking, it affects the drying rate and efficiency. Higher inlet air temperatures lead to a faster drying rate and shorter residence time, but at the same time, they increase the risk of thermal degradation and the loss of volatile components. Therefore, for target natural components that are easily decomposed or thermally degraded, their inlet air temperature should be strictly controlled or freeze spray drying should be used; outlet air temperature generally refers to the temperature of the dry gas leaving the drying chamber, which reflects the heat and moisture transferred from feed to air during the drying process. It affects the product’s moisture content and stability. Generally speaking, lower temperatures mean significant heat exchange between gas and liquid, low humidity content, and high stability; when target natural extracts are extremely sensitive to high temperatures or humid heat, freeze spray drying can be used instead of conventional spray drying (103, 104). The drying process of this method includes three main steps: droplet formation, freezing, and sublimation drying. The drug and dispersion medium are rapidly cooled and distributed in frozen droplets, then sublimated to form amorphous, porous particles (105–108). (5) Peristaltic pump speed: Also known as feed rate, it is the rate at which feed solution or suspension is delivered to the atomizer. It affects droplet size distribution, drying rate, and product residence time. Generally speaking, a higher feed rate leads to larger, more uneven droplets, a slower drying rate, and a longer residence time, which may affect product quality and yield. (6) Atomization pressure: the pressure used to force feed through the atomizer nozzle. This pressure comes from various sources, such as high-pressure gas, centrifugal force, etc., and directly affects droplet size distribution and atomization efficiency, thus affecting product particle size distribution. Generally speaking, higher atomization pressure leads to smaller, more uniform droplets, resulting in a more uniform particle size distribution with a smaller average particle size.

For natural product DPI preparation, according to its instability, low solubility, and bioavailability characteristics, reasonable adjustment of the above parameters can obtain the best overall performance products.

### 3.1. Types of DPI powders

Depending on the characteristics of the active pharmaceutical ingredients (API) and the demand for the formulation, we can adapt the spray drying process to prepare granular products in different forms and states, such as common nanoparticles, microspheres, liposomes, and porous nanoparticles (109–111). The different forms of particulate products offer different advantages and have been tailored to the different clinical needs of the products; for example, nanoparticles allow targeted delivery of drugs independent of the physiological environment and facilitate transport across biological barriers (112); microspheres are used to reduce the adhesion of drug carriers, which greatly increases the fraction of fine particles (FPF) (113); and liposomes are used to avoid immune rejection, select drug localization in the lungs, and reduce local or systemic toxicity (114). Three widely used dosage forms are discussed below.

#### 3.1.1. Nanoparticle

Nanoparticle DPIs are made by mixing drug-loaded nanoparticles with a suitable matrix carrier and using spray drying technology to produce inhalable particles. The use of nanospray dryers allows for the production of particles with a more uniform size and a smaller particle size, thus further improving the bioavailability of inhaled powdered drugs. It has been shown that preparing curcumin-loaded casein nanoparticles by spray drying sodium casein (NaCas) and curcumin in an ethanol solution improves the dispersibility of the drug. Curcumin encapsulated in casein nanoparticles has higher bioactivity than pristine curcumin, as assessed by antioxidant and cell proliferation assays (115). The nanoparticles also have good dispersion properties, lung deposition properties, and both slow and controlled release (116). Dimer et al. prepared resveratrol by piezoelectric atomization technique and nanospray drying. They obtained spherical particles with irregular surfaces, low water content (less than 2.0%), low density, high mobility, and good dispersion. This nanoparticle drug deposition is suitable for the bronchial and alveolar regions of the targeted lung, achieving better therapeutic effects (117). Moreover, nanoparticles can be surface-modified so that they are not cleared by macrophages *in vivo*. This prolongs the contact time of systemic circulation, enhances the therapeutic effect of the drug, and extends the retention time of the drug in the body, thus prolonging the therapeutic effect (118). In addition to this, studies have also been conducted to achieve sustained drug release by preparing nanoparticles as polymers and using the solubilization and adhesion properties of polymers (119).

#### 3.1.2. Microspheres and microcapsule

Microspheres, or microcapsules, are skeletal microspheres formed by dissolving or dispersing a drug in a polymeric material. They both refer to particle dispersion systems formed by dispersing or adsorbing a drug in a polymer. Microspheres are characterized by their large particle size and various morphologies, such as spherical, oval, and rod-shaped. Due to the large specific surface area and pore structure of microspheres, drugs can be embedded and adsorbed inside them, achieving sustained drug release through slow release (120). When manufacturing dosage forms using spray drying technology, drugs are dissolved in an organic solvent to form an emulsion, which is then spray-dried using a spray dryer to obtain microspheres or microcapsules with uniform particle size. Encapsulation of drugs does not lead to loss of activity or denaturation of biologically active substances but rather improves their stability and gives them a sustained release effect. Jovanovic et al. (121) obtained microspheres with higher thermal stability and a higher diffusion coefficient by spray drying. Desai et al. (122) used spray drying to prepare vitamin C-coated chitosan microspheres cross-linked with tripolyphosphate (TPP). Follow-up studies confirmed that vitamin C release from TPP-chitosan microspheres was sustained and that vitamin C was more stable than pure vitamin C. One study prepared anthocyanin-rich microcapsules by spray-freeze-drying a complex condensed double emulsion. The microencapsulation efficiency (MEE) of the microcapsules changed from 84.9 to 94.7%, with higher total anthocyanin and total phenol content. Their sustained release properties were found by testing their *in vitro* release characteristics, reflecting their good performance in applications. They can provide a good clinical solution for some drugs that have a short half-life in circulation and are easily degraded (123).

#### 3.1.3. Liposome

Liposomes are closed vesicle materials similar to the biological membrane structure formed by encapsulating active substances using liposome technology with a phospholipid bilayer biological membrane structure. Liposomes are composed of amphiphilic molecules, and both water-soluble and fat-soluble drugs can be encapsulated. The fat-soluble drugs are positioned between the bilayer lipid membranes, and the water-soluble drugs are wrapped in the water phase (124). Liposomes can improve the stability of drugs. Because drugs are wrapped in liposomes before entering the target area, they are not decomposed by body enzymes and other factors (111). Curcumin precursor liposomes prepared in a research study using a spray dryer showed enhanced growth inhibition of lung tumor cells A549 and a significant reduction in associated cytokines such as tumor necrosis factor-, interleukin-6, and interleukin-10 compared to the pure drug. Results from pulmonary pharmacokinetic assays confirmed the superiority of curcumin precursor liposomes over curcumin powder in terms of the rate and extent of lung tissue uptake and the mean retention time in lung tissue (5). In contrast, Xu et al. prepared a spray-dried powder of vincristine (VCR) liposomes, and the aerosol had better pharmacokinetic behavior compared to free VCR: Compared to free VCR, its maximum concentration and systemic exposure, respectively, increased by 630.8 and 429.6%, and its elimination half-life was shortened by 81.1%, while the clearance rate was reduced by 83.2%, demonstrating the excellent facilitation of drug bioavailability by spray-dried DPIs (125).

## 4. DPI principles and advantages

### 4.1. Principles of DPI

Dry powder inhalers are popular because they offer several advantages over other types of inhalers, such as metered dose inhalers (MDIs). The following describes the operation of dry powder inhalers. Dry powder inhalers can be divided into the following types, depending on the form of the drug and how it is released: (1) Metered-dose dry powder inhalers: These inhalers release a certain amount of drug each time and usually require pressing or twisting the device to activate the drug. Examples include Advair Diskus, Flovent Diskus, and Serevent Diskus; (2) Breath-activated dry powder inhalers: These inhalers release the drug by the user’s breathing force, without the need to press or twist the device. Examples include Symbicort Turbuhaler, Asmanex Twisthaler, and Pulmicort Flexhaler. However, note that this type of inhaler may not be suitable for patients with respiratory dysfunction; (3) Single-dose dry powder inhalers: These inhalers can only be loaded with one dose of medication at a time and require the insertion of a capsule or disc before each use. Examples include Spiriva HandiHaler and Foradil Aerolizer.

All three types of inhalers share a common principle. First, the drug formulation: DPIs use a dry powder formulation containing the active drug. The drug is usually micronized to ensure that the particles are small enough to penetrate the lungs. Then, respiratory drive design: DPIs are usually respiratory drive devices. Drug storage and dose preparation: The DPIs have a drug reservoir containing multiple doses of drug. Each dose is then individually packaged or prepared in the device to ensure proper dosing. Some DPIs use capsules, while others use pre-packaged blisters or spacers that the patient inhales at the same time as the drug is released for therapeutic purposes.

Different types of dry powder inhalers have different advantages and disadvantages and are used in different ways. The researcher or patient may choose the appropriate type of dry powder inhaler based on clinical facts or personal preference.

### 4.2. Advantages over other traditional delivery methods

The advantages of dry powder inhalers over traditional methods of drug delivery, such as oral administration and injections, are obvious, especially for specific drugs, diseases, and patients. For example (126–131):

(1) Direct action on the lesion: dry powder inhalers can act directly on the lungs through the respiratory tract, delivering the drug directly to the lesion, reducing the metabolism and side effects of the drug in the body.
(2) Rapid onset of action: dry powder inhalers can be quickly absorbed into the alveoli, and then quickly enter the blood circulation, so that the drug effect works quickly.
(3) High patient compliance: especially for some patients with swallowing difficulties, such as the elderly, children, and patients with digestive tract diseases, or some drugs containing bitter or offensive tastes.
(4) Small amount of drug use: Compared to oral drugs, dry powder inhalers require a small amount of drug use, which can reduce the accumulation of drugs in the body and reduce the occurrence of toxic side effects of drugs.
(5) Convenient and easy to use: dry powder inhalers can be used at any time and any place, easy to use and not disturbed by the external environment, especially suitable for long-term treatment of patients with chronic diseases.
(6) Avoid liver first pass effect and enterohepatic circulation, increasing the bioavailability of the drug.
(7) Environmentally friendly and pollution-free: dry powder inhalers do not require solvents or gases as carriers and do not produce waste or harmful substances in the process of use, which is less polluting to the environment and safer and more environmentally friendly than other drug delivery methods.

### 4.3. Advantages relative to other pulmonary drug delivery methods

And the advantages over other methods of lung drug delivery are also very obvious. DPIs are one of the four main devices used to deliver pharmaceutical preparations to the lungs, along with pressurized metered dose inhalers (PMDIs), nebulizers and soft mist inhalers (SMIs) (23, 132). DPIs also offer several advantages over the other three liquid drug delivery devices:

#### 4.3.1. Environment-friendly

Pressurized metered dose inhalers were once the most widely used inhalers, but they use chlorofluorocarbon (CFC) propellant in the atomization process to deliver to lung tissue, which can lead to depletion of the ozone layer. Dry powder inhalers, on the other hand, are administered as a dry powder and do not require propellant to deliver the drug into the lungs. This makes dry powder inhalers more environmentally friendly and their reusable nature offers significant advantages in terms of resources and energy consumption.

#### 4.3.2. Availability

Nebulizers in in-hospital formulations are compressor driven, which is heavy, bulky, noisy and has limited portability compared to dry powder inhalers (81, 133). On the other hand, dry powder inhalers are portable, do not require an external power source or propellant and can be carried around with the medication according to the patient’s needs, making them more convenient for the patient to use (134, 135).

## 5. Application and development of natural extracts DPI

Studies have shown that spray drying can significantly improve the efficiency of drug absorption in the lung, improve drug distribution, avoid drug deposition, increase its bioavailability, and allow natural products to exert their target clinical effects (136, 137). For example, in amorphous formulations obtained by spray drying ferulic acid with 10% or 20% PVP-K30, PEG 6000, or Poloxamer-188, free radical scavenging assays confirmed complete antioxidant activity and increased antiplatelet effects.

According to BBC Research, the global pulmonary drug delivery systems market totaled USD 38.1 billion in 2017 and is expected to reach USD 47 billion by 2022, growing at a compound annual growth rate (CAGR) of 4.3% during 2017–2022. The huge market demand is aiding the research on spray drying of natural ingredients for dry powder inhalation. The application of dry powder inhalation has been widely used in formulations for the continuous delivery of atomized particles. Spray drying technology allows the production of free-flowing powders of various sizes, offering unlimited possibilities for the production of dry powder for inhalation.

### 5.1. Improving the target organ concentration of drugs

Dry powder inhalers exhibit good aerosol properties and can effectively deliver the drug to the lungs and obtain high pulmonary drug concentrations. Hu et al. (138) found that the production of curcumin-DPI by wet milling combined with spray drying resulted in significantly higher plasma curcumin concentrations after inhalation, and most of the curcumin-DPI was deposited in the lungs with high target organ drug concentrations and good efficacy. In addition, Meenach et al. (139), by spray drying an organic solution composed of paclitaxel (PTX) liposomes, found that DPIs with a product median diameter (MMAD) between 1.9 and 2.3 μm, good aerosol dispersion, and a high deposition rate in the lungs were effective in improving the therapeutic effect and achieving better therapeutic goals. DPIs not only increase the local concentration of drugs and achieve targeted distribution of drugs, but also reduce the distribution of drugs in other organs or tissues, reduce or avoid the adverse effects of certain natural ingredients in non-targeted sites, and improve the effectiveness and safety of natural active target substances.

### 5.2. Improving bioavailability

Since the bioavailability of most natural ingredients is low, an important application of DPI is to improve this problem. DPI is administered directly through the lungs without passing through the enterohepatic circulation, avoiding the first-pass effect and allowing increased entry into the systemic circulation, thus improving bioavailability. Lu et al. (140) prepared salvianolic acid (SMPA) by spray drying and found that, compared to oral administration, pulmonary administration of salvianolic acid B (Sal B) had a bioavailability of up to 19.15%. This strongly suggests that target natural bioactive ingredients with low bioavailability can be effectively applied by spray-drying DPI, which is important for some active ingredients with poor effects in animal experiments but significant pharmacological effects in cellular experiments, such as plant extracts with short half-lives, easy metabolism, and low solubility, which may bring new opportunities for these drugs that would be prematurely eliminated because of these drawbacks.

### 5.3. Improved stability

In dry powder inhalers, the drug is stored as a dry solid powder; the drug is less susceptible to environmental disturbances during production, transport, and storage; the molecular activity is low; the drug is protected from oxidation and decomposition; and its physical and chemical stability is improved. In addition, stabilizers are usually introduced in the formulation design to ensure that the active ingredients of the drug are not destroyed. One study (112) found that formulations of budesonide (BUD) and colchicine (COL) liposomal DPI were stable for 6 months at 25 ± 2°C, 60% RH, and refrigerated conditions (2–8°C) compared to conventional formulations. The stability of the formulations was greatly improved, and the stability of the active ingredients was effectively ensured. Similarly, DPI is a better choice for natural active ingredients with poor stability.

Dry powder inhalers for inhalation of various natural ingredients are described in Table 3.

**TABLE 3:** Dry powder inhalers with natural ingredients (141).

Names	Characters
Curcumin microspheres	Sustained release in Raw 24.264 cell line for up to 7 h with satisfactory *in vitro* cytotoxic activity (142)
Curcumin micelles	2.20 and 1.77 fold increase in bioavailability and Cmax (138)
Curcumin nanoparticles	Nearly fourfold increase in solubility (143)
Andrographolide	ED and %FPF increased 1.84-fold and 1.47-fold, respectively, and bioavailability increased 1.73-fold (144).
Fisetin	FPF is increased by 2.3 times, improving the water solubility of laccasein and delivering large amounts of laccasein to the deep lung region for treatment (67).
Salvianolic acid B (SALB)	Increased bioavailability and its drug concentration in the lungs (145).
Extraction of *Trollius chinensis*	Fine particle fraction (FPF) and emission dose (ed) were (21.07 ± 1.74)%, (75.31 ± 21.05)%, respectively (146)
*Panax notoginseng* saponins, *Salvia miltiorrhiza*	FPF close to 60% (147).

## 6. Conclusion and perspective

In contemporary drug formulation design, it is very important to find the appropriate active ingredients, dosing route, and production process for the reasonable application of natural foods with medicinal value. Many natural resources are of rich medicinal value, and natural ingredients are often used as the main raw materials for drugs. However, their unstable chemical properties, low solubility, and low systematic bioavailability often hinder their development and application. Therefore, there is an urgent need to find suitable preparation methods and application means. In this paper, the principle and synthesis of spray drying technology in the preparation of dry powder inhalers of natural products are reviewed, and some successful applications are analyzed. In a word, the use of spray drying technology to produce drugs containing natural ingredients has great prospects and provides the possibility for more drug applications of natural products. In the future, with the increasing incidence of lung diseases such as lung cancer, pneumonia, chronic obstructive pulmonary disease, and pulmonary fibrosis, preparations for pulmonary administration, especially dry powder inhalers, will have great research significance and market prospects. Especially with natural active products, their reasonable and scientific use is also a major breakthrough in pharmaceutical research. Therefore, the research and application of dry powder inhalants with natural active products are important for human health and scientific development.

## Author contributions

BW: writing—original draft preparation. JX, ST, and WZ: writing—review and editing. ST and BH: supervision and approval. All authors contributed significantly to the writing of the manuscript.
